# The Narcissist Next Door. Understanding the Monster in Your Family, in Your Office, in Your Bed – in Your World

**DOI:** 10.1192/pb.bp.115.053124

**Published:** 2017-04

**Authors:** Jon Patrick

I find myself split in my thinking about this book. On the one hand, I can see its appeal as an airport read; requiring little effort to get through, and full of celebrity and political commentary as well as easily digestible chunks of scientific evidence.

At that level, it's enjoyable. Especially so when it allowed me to neatly project all my ugly narcissism into reports about Kanye West and Sarah Palin. Perhaps a first for them to be mentioned in the *Bulletin*, no doubt adding to their narcissistic satisfaction, should they or their agents be subscribers.

At another level – and this is where I'm split – it is an exercise in quite contemptuous character assassination. Kluger's portrayal of his example subjects is cold and sneering at times. Furthermore, he often seems to conflate the concepts of narcissism and psychopathy, leading to a sense that the more narcissistic of us are one step away from becoming serial killers or workplace tyrants.

There are only brief mentions of how the presentation of narcissism might be related to inner vulnerability, and this left me wondering if Kluger might have been looking at the mirror crack'd. Even as I write this I wonder if I too am succumbing to the narcissistic appeal to feel superior to what we read – this is hard to contain when I am a UK reader and the author mentions former prime minister ‘Malcolm Browne’ (referring to Gordon) and the football team ‘Aston Vista’. Such mistakes feel sloppy, arousing my narcissistic contempt; perhaps a response to feeling as though the author does not care enough about the UK to check facts properly.

Coming from a psychoanalytic tradition, where this subject has been a preoccupation of clinicians since Freud's 1914 *On Narcissism: An Introduction*, Kluger's view on the dilemma of the narcissist saddened me. We are all narcissists to some degree; it's what allows us to get out of bed in the morning and feel like we are good people who might be loved. The pathological narcissist is someone who has found their early experiences to be lacking and who has lost their trust in acceptance by others. To manage this insufferable situation, they create an outer self that is contemptuous of need and full of itself, and project away their dependent, vulnerable selves onto others. Sometimes, they are contemptuous and dismissive of needy people. Sometimes, if society is lucky, and the person more creative, they will look after others who are vulnerable – to repair the damage they feel inside themselves.

